# Value chain organization models of Taiwanese electronic information enterprises in Mainland China and their spatiotemporal evolution processes

**DOI:** 10.1371/journal.pone.0254402

**Published:** 2021-07-22

**Authors:** Chen Siyue, Wei Suqiong, Huang Gengzhi, Zhang Hongou

**Affiliations:** 1 Tourism College of Hainan University, Haikou, China; 2 Department of Human Geography and Regional Development, Guangzhou Institute of Geography, Guangzhou, China; 3 College of Geographical Sciences, Fujian Normal University, Fuzhou, China; 4 College of Geographical Sciences, Sun Yat-sen University, Guangzhou, China; Institute for Advanced Sustainability Studies, GERMANY

## Abstract

This study examines Taiwanese investment in Mainland China as it is an important part of cross-strait economic cooperation. Using sample data from Taiwanese-listed electronic information enterprises in Mainland China (1990–2016), this study combines ArcGIS spatial visualization and case analysis to investigate their value chain organization models and spatiotemporal evolution regularity. The results show that the value chain of the electronic information industry for Taiwanese investments in Mainland China has three models: vertical integration, modularization in production sharing, and production extension. Vertical integration is the main production organization model of these Taiwanese listed electronic information enterprises, expanding from single production to the entire manufacturing value chain, followed by sales, and finally R&D. This model is still in use in the Western Taiwan Straits Economic Zone, whereas the other four Taiwanese investment agglomerations, namely the Bohai Economic Rim, Yangtze River Delta, Pearl River Delta, and Western Delta Economic Circle, began to expand to both ends of the production link, particularly to the sales link. High-value -added enterprises adopting production sharing models began to show a trend of expansion to inland cities, and enterprises adopting the manufacturing–sales model (a production expansion model) had the widest distribution. Finally, at the city level, the value chain fragmentation structure of Taiwanese and developed countries’ cross-border (multinational) enterprises in Mainland China were consistent, that is, they matched the Chinese city hierarchy; at the regional level, however, the Western Delta Economic Circle pioneered to become a hub for Taiwanese electronic to information enterprises set up their R&D and sales links in Mainland China. Investigating chain-alike spatiotemporal expansion of Taiwanese investment in Mainland China is important for the integration and development of the value chain, production network, and enterprise spatial organization theories.

## Introduction

Since 1978, Mainland China has adopted open market and devolution strategies as part of its economic reform program. Attracted by incentives from local governments and low-cost labor and land, foreign direct investments (FDIs) have flooded Mainland China. As original equipment manufacturers (OEMs), high-tech Taiwanese enterprises have followed their global contractors and shifted their production bases to Mainland China. In recent years, high-tech Taiwanese enterprises have changed the way they invest in Mainland China to adjust to its economic structure and changes in the investment environment. Their value chain systems have transformed from a single production process to the integration of R&D, manufacturing, and sales. Exploring the value chain organization models of the Taiwanese high-tech industry in Mainland China as well as their spatiotemporal expansion processes and evolution trends will allow the various regions of Mainland China to better formulate policies for Taiwanese enterprises and be in a better position to benefit from positive spill-over effects derived from high value-added links.

Existing research on Taiwanese investment in Mainland China has focused on investment motives and prospects [1, 2], spill-over effects [3–5], and whether Taiwanese investors adopt different site selection strategies compared to other foreign direct investors in China [6–10]. Some researchers have also discussed the spatial organization structure of Taiwan’s information technology industry network in Mainland China, evaluated the significance of cross-strait R&D for global production networks [11–13], and examined the evolution of clusters, technology catch-up processes, and trends in Taiwan’s integrated circuit industry in Mainland China [14, 15]. Others have explored the interweaving and regional strategic coupling of Taiwanese laptop manufacturers’ cross-border expansions and have emphasized the importance of institutional arrangements with the national government and interactions with local enterprises in shaping local development [16–20]. These studies have laid the foundation for relevant research on Taiwanese high-tech industries in Mainland China. However, they have not addressed that the development of Taiwanese high-tech industries in Mainland China is not only an issue of spatial agglomeration and coordinated regional development, but also of the spatiotemporal expansion process of the value chain.

To fill the gap in the literature and understand the value chain organization models of the Taiwanese electronic information industry in Mainland China and its spatiotemporal evolution processes, we analyzed Taiwanese electronic information enterprises with the longest investment history and largest number of subsidiaries in Mainland China (1990–2016). ArcGIS spatial visualization and case analyses were applied to examine the chain-alike spatial expansion process, extent, and patterns of Taiwanese investment in Mainland China from the perspective of the electronic information industry value chain.

This paper is organized as follows: Section 2 reviews the related literature, Section 3 explains the data sources and research methods, Section 4 presents the results, and Section 5 concludes.

### Literature review: Value chains (VCs) and global value chains (GVCs)

**Fig 1:**
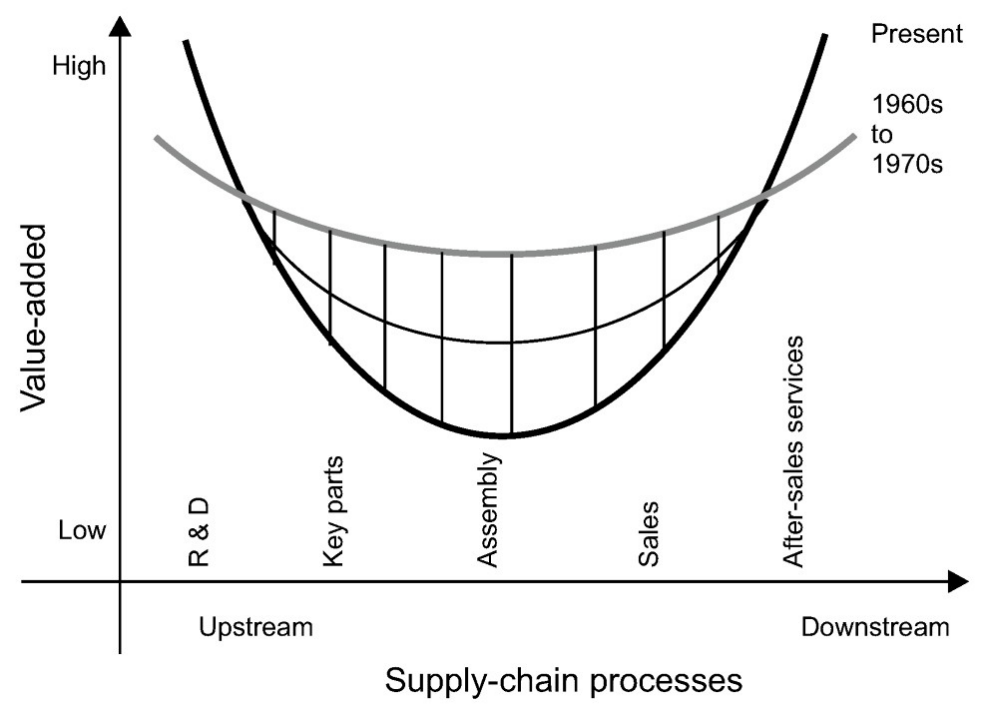
Spatial positioning map for the value chain models of enterprises (2016).

The concept of VCs, as both a theory and a methodology, first arose from the work of Michael Porter in the mid-1980s. Porter defined a value chain as the chained linkage of related and dependent activities for delivering value from product design to final customs; this involves design and production development, productive processes, marketing, and consumption recycling at the firm level [21]. Essentially, value chain analysis of value-added stages refers to two dimensions: “internal” VCs, from purchasing materials to distributing, selling, and servicing the final product within a company; and “external” VCs, from raw materials to manufacturing and distribution of the product among end-users, with each stage representing an industry [22, 23]. In 1992, Acer founder, Stan Shih advanced “the smiling curve” with an analysis of the computer industry’s development [24] (Fig 1), and explained that the level of power exertion and value capture is not equally spread among participants in different stages of the chain. Specifically, upstream activities consist of basic and applied research and development, whereas downstream activities typically comprise marketing, advertising, brand management, sales, and after-sale services. The lowest value-added activities are assembly, manufacturing, and other repetitive processes that are in the middle of the VCs and are the easiest to outsource. Subsequently, VCs have expanded globally, through general business activities including assembly, OEM, original design, manufacturing, and own brand manufacturing. Accordingly, research has analyzed cross-border manufacturing and distribution systems as well as the whole supply chain. GVCs emphasize how large and powerful leading firms and contract manufacturers bridge functional linkages and coordinate internationally dispersed activities to achieve dominance in the global market [25, 26].

#### GVCs’ organization model and its geographical restructuring

**Fig 2:**
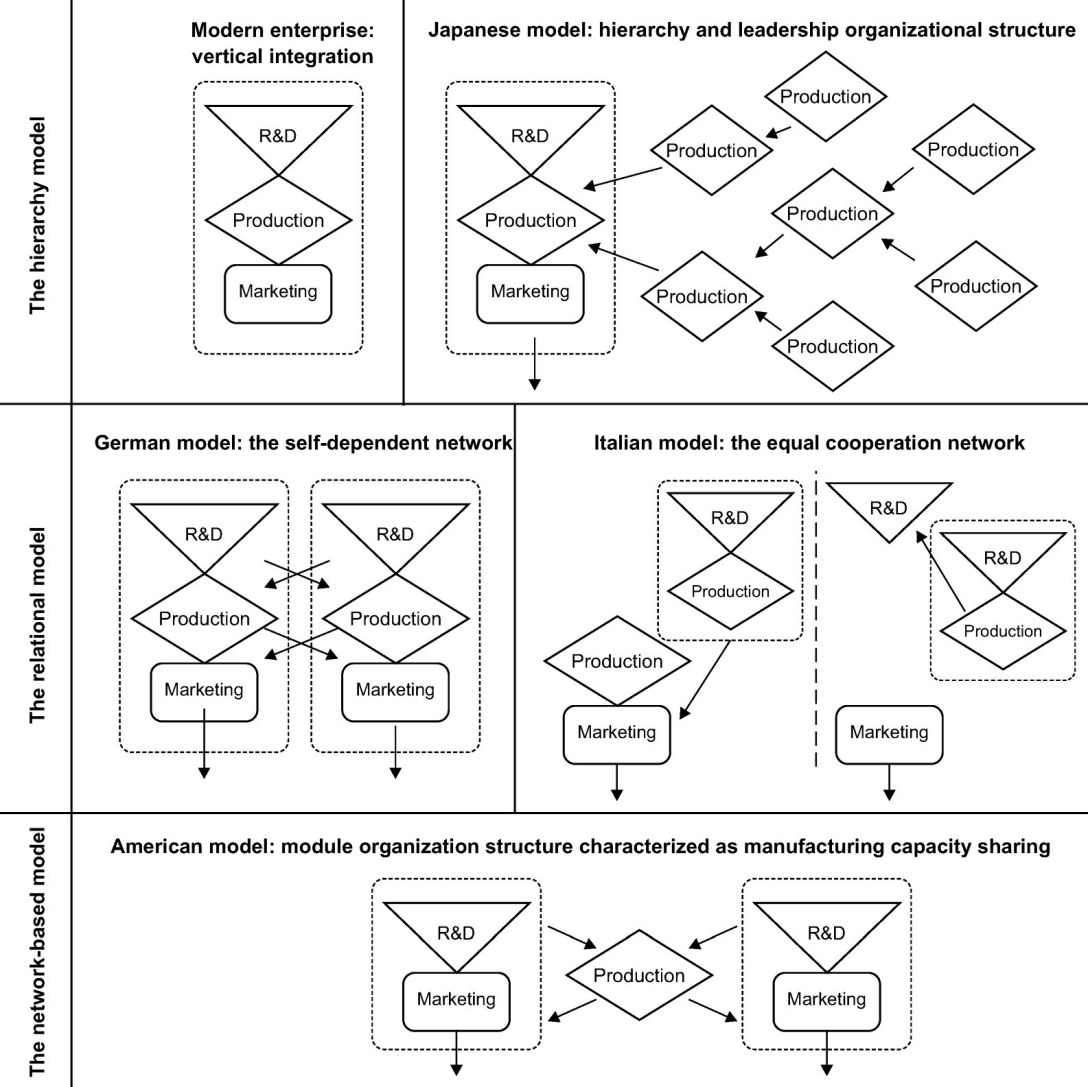
The organization mode of multinational company production networks. Spatiotemporal distribution of the value chain organization models of Hon Hai Precision Industry, Wistron Corporation, and AsusTeK.

Since Krugman et al. [27] discussed the interaction between “slicing up the value chain internationally” and industry clustering and noted the ability of reorganizing various value chain nodes of production processes within a firm in different locations, it has been generally accepted that the level of power exertion and value capture are not evenly spread among participants in different stages of the chain or in the same regions. Particularly, the increasing significance of a modular theory-building effort to explain value chain organization governance has been intensively emphasized in recent years [28–30]. This topic’s literature has scrutinized industrial/firms’ VCs from the organizational modularity perspective with Sturgeon [31] and Gereffi et al. [28] contributing much to this effort. These authors drew on the theory of modularity and their understanding of VCs to define “value chain modularity” and “modular production network” as terms for the new industrial organizational form that emerged in the US electronics industry between the late 1980s and early 1990s. They proposed three organizational models for the production network of multinational companies—hierarchical, relational, and network-based—which have drawn extensive attention in academia (Fig 2). Specifically, the hierarchical model refers to the modern enterprise’s vertical integration organization structure and the level and leadership organization mode represented by Japanese and South Korean enterprises, which focus on production, especially on core component production parts, final product assembly, and the outsourcing of non-core manufacturing activities. The relational model, in which social relations play an important role, can adapt to flexible and changeable market demand and is represented by Italian and German enterprises. Lastly, the network-based model refers to the module organization model characterized by manufacturing capacity sharing, as is the case in American enterprises; leading enterprises (brand owners) focus on product design, marketing, and distribution while completely outsourcing the manufacturing process to contract manufacturers.

From a theoretical perspective, more effort on how to embed in GVCs and their impact on industrial clustering and upgrading has been made by some scholars. For example, Humphrey and Schmitz [32, 33] examined the link between industrial agglomerations and GVCs’ governance, identified how global industrial participators, especially developing country producers, insert into GVCs by proposing the four paths of “arm’s length market relations, network, quasi-hierarchy, hierarchy,” and further analyzed how the four kinds of insertion in GVCs affect the upgrading of industrial clusters with regard to different types of upgrading (e.g. process upgrading, product upgrading, functional upgrading, and interpectoral upgrading).

From a practical perspective, the pattern of geographic distribution and regional division of labor on some industrial VCs, such as clothing and footwear [34–37], furniture [38, 39], cars and bicycles [40, 41], and electronics [42–45], has been analyzed. These studies confirm that as GVCs emerge, the production organization mode of transnational enterprises undergoes significant changes such that it is no longer manifested only as a single vertically integrated organization feature; the enterprises contain each value chain link of R&D, manufacturing, and sales.

However, as the industry matures and develops, the governance model of the value chain may change [46]. Sturgeon et al. [44] stressed that it is important not to ignore the need to understand the spatial processes and the various roles that local agglomerations play within spatially extensive VCs. Furthermore, the authors identified the need to map activities that tend to be concentrated in specific places even as the geographic “footprint” of linked economic activity expands. In other words, these empirical research papers conducted static analysis, and there is a lack of literature on the spatiotemporal patterns of industrial VCs. More specifically, there is a gap in the literature regarding the dynamics and evolutionary processes about when, where, and how industrial value is created. Therefore, we assess the spatiotemporal evolution processes of industrial VCs. The specific focus of our investigation is Taiwanese-funded ITE industry VCs in Mainland China. On this basis, we generate new research conclusions in this field.

#### GVCs and Mainland China

At present, there are three approaches to research about Mainland China’s value chain. The first is to quantitatively measure the status and participation degree of China’s manufacturing industry in the GVC from three perspectives: overall, by segmenting industries, and by comparing internationally. The research results show that, on the whole, China’s manufacturing industry has a high degree of participation and a relatively low position with respect to the division of labor, but with an upward trend observed for the latter [47–51].

The second is based on an economic perspective. In this approach, relevant theories of value chain upgrading are used to analyze the upgrading path and driving mechanism of specific industries or specific value chain links [52–54]. The generation and upgrading modes are divided and summarized from different perspectives, such as the path selection of technology research and development and application, the development characteristics of specific industries, the relationship between industrial upgrading and production factors, and the direction of the product market [55, 56].

The third approach analyzes the geographical distribution and spatial strategies of specific industrial VCs from a spatial perspective [57–62]. However, research based on different spatial scales, for example, transboundary production networks implanted in different countries and small and medium-sized enterprise clusters, should be supplemented. With the newly industrialized countries (or regions) building cross-border production networks in the economically underdeveloped countries (or regions) independently, the organization and spatial reconstruction of the value chain phenomenon has become increasingly common among Taiwanese business people through investment, especially since the 1990s. For example, the electronic information industry has played an increasingly important role in promoting the economic and social development of both sides of the Taiwan Straits by integrating into the global production network and building the sub-global production network independently. In recent years, with changes in economic structures and the investment environment in Mainland China, the investment mode of Taiwan business people has been constantly adjusting. From the point of view of the value chain system, the organizational models of Taiwan-funded enterprises in Mainland China range from single processing and manufacturing to research and development, manufacturing, and sales synchronous development.

In this context, we choose Taiwanese-funded ITE industry VCs in Mainland China as the research objects, the value chain organization models of the Taiwanese electronic information industry in Mainland China, and its spatiotemporal evolution of the processes. The main contributions of this study are as follows: first, we present a comprehensive understanding of the spatial correlation mode, organizational pattern, and evolution process of the value chain of Taiwan-funded electronic enterprises in the mainland to provide decision-making support for the future spatial layout, planning, and development strategies of Taiwan-funded economies on both sides of the Taiwan Strait. Second, we clarify the status and representation of each region in the Taiwan-funded electronic information industry to provide an understanding of the relevant strategies of mainland China, including integrating into the global production network and independently building a secondary global production network.

## Materials and methods

Taiwanese electronic information manufacturers with more than 10 subsidiaries in Mainland China and listed in Taiwan were selected because the value chain models of those enterprises were relatively complete and therefore conducive to the screening and development of subsequent case studies.

Names, addresses, registration dates, and business scopes of 542 Taiwanese electronic information enterprises with more than 10 subsidiaries in Mainland China were obtained from the List of Taiwanese Listed (Over-the-Counter) Enterprises in Mainland China (1990–2016). Data reliability was verified through each enterprise’s official website and missing information on business scope and addresses were added. Of those enterprises, 24 with a classified business scope marked were excluded, leaving 518 for analysis. Next, the locations of each enterprise’s value chain links were identified using the API interface provided by Google Earth to map out a relatively complete statistical information base of the spatial organization of the enterprises. The map identified 76 administrative districts above the prefecture level as spatial units. The spatial analysis software ArcGIS10.1 was used to import the geographical and spatial correlation network data of enterprises in each value chain link and to analyze the spatial attributes of network characteristics of Taiwanese enterprises.

Based on business scope and corporate organization, we divided the value chain of Taiwanese-listed over-the-counter (OTC) electronic information enterprises into three links: R&D, manufacturing, and sales and service. Particularly, the businesses involved in the R&D link of Taiwanese electronic information enterprises could be divided into two types: 1) the development of non-core components of electronic computers and their peripheral devices, including large- and medium-sized computers, portable computers, electronic components, electronic-dedicated equipment, instrument components, integrated circuits, and other basic designs; and (2) the aesthetic design of electronic industrial products and the structural design of mechanical parts, including car navigation, photographic equipment, game consoles for smart home appliances, mobile phones, and spare-parts servers.

## Results

### Value chain organization models of Taiwanese electronic information enterprises

**Fig 3:**
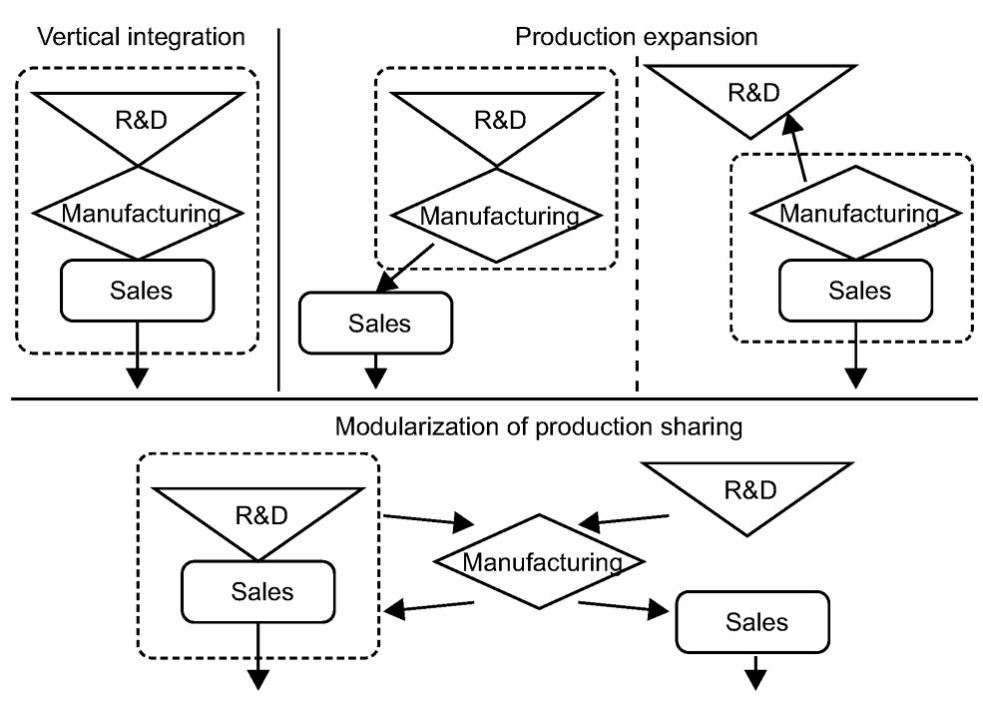
Value chain organization models of Taiwanese OTC electronic information enterprises in Mainland China.

Sturgeon [31] proposed three organizational models for the production network of multinational companies: hierarchical, relational, and network-based (Fig 2). The hierarchical type refers to the vertically integrated modern enterprise model and the leading organizational model represented by Japanese and Korean companies. This type often focuses on the production process, especially the production of core components and the assembly of the final product. The non-core manufacturing links and parts productions are outsourced. The relational type includes organizational models represented by Italian and German companies, in which social relations play an important role. The network type refers to the modular organization model represented by companies in the United States, which share manufacturing capacity and focus more on R&D and brand marketing. Taiwanese OTC electronic information enterprises in Mainland China typically also have a vertical integration model and two categories comprising six other value chain organization models: 1) production sharing modularization, including R&D, production, sales, and R&D–sales; and 2) production expansion, including manufacturing–sales and R&D–manufacturing (Fig 3).

**Fig 4:**
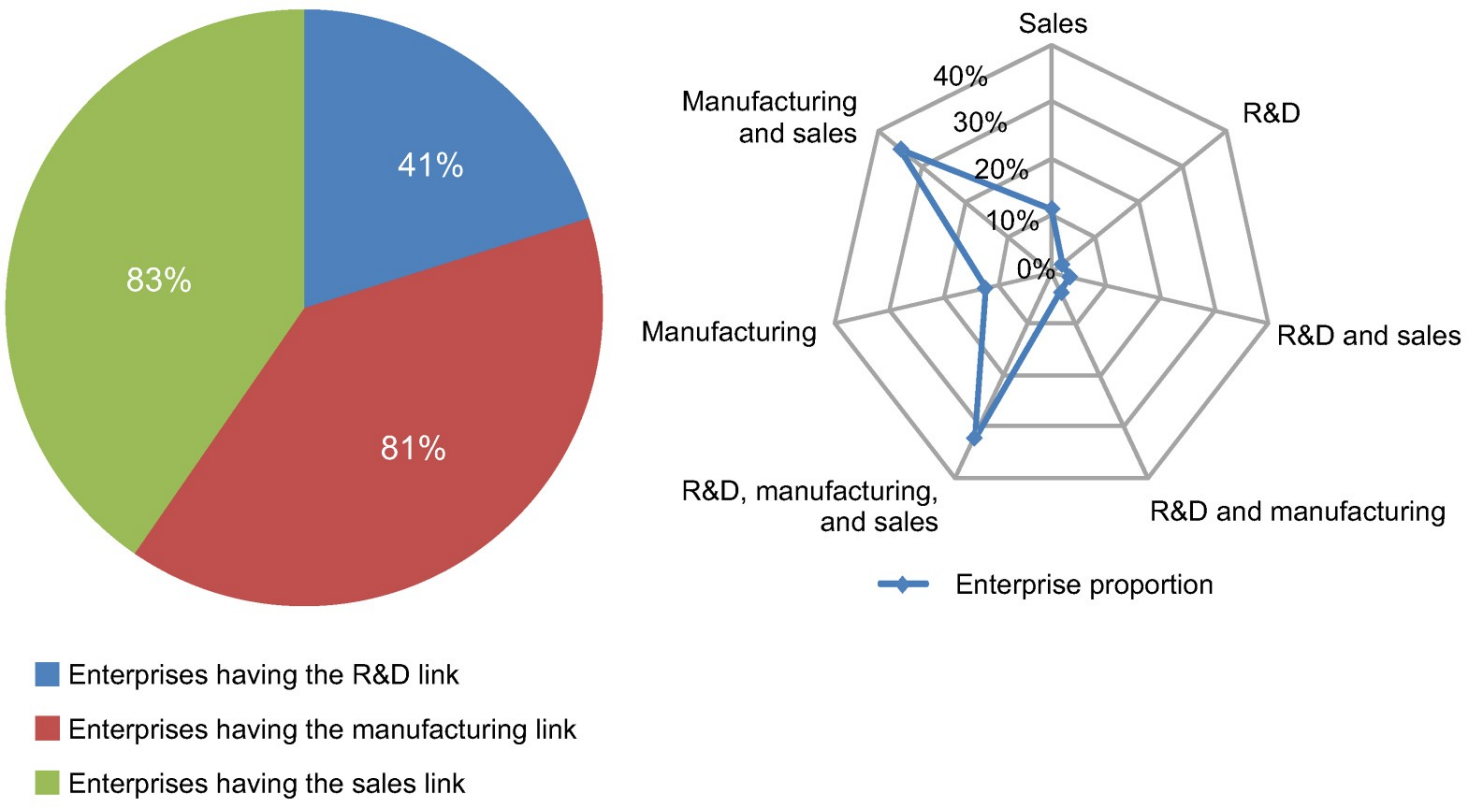
Proportions of Taiwanese-listed OTC electronic information enterprises for each value chain organization model.

Fig 4 shows the proportion of Taiwanese OTC electronic information enterprises for each value chain model and each value chain link, respectively. The figure indicates that most enterprises adopted the vertical integration model or the manufacturing–sales model, with more than 30% of the Taiwanese enterprises adopting the model. Although some enterprises had begun to focus on the R&D link of electronic components, their main operations were still manufacturing and sales. For example, the proportion of enterprises with a manufacturing link was the largest, reaching 83%, followed by enterprises with the sales link (81%), both of which were about twice as large as the proportion of enterprises with R&D links (41%).

### Spatiotemporal evolution characteristics of value chain organization models

#### Macro level

**Fig 5:**
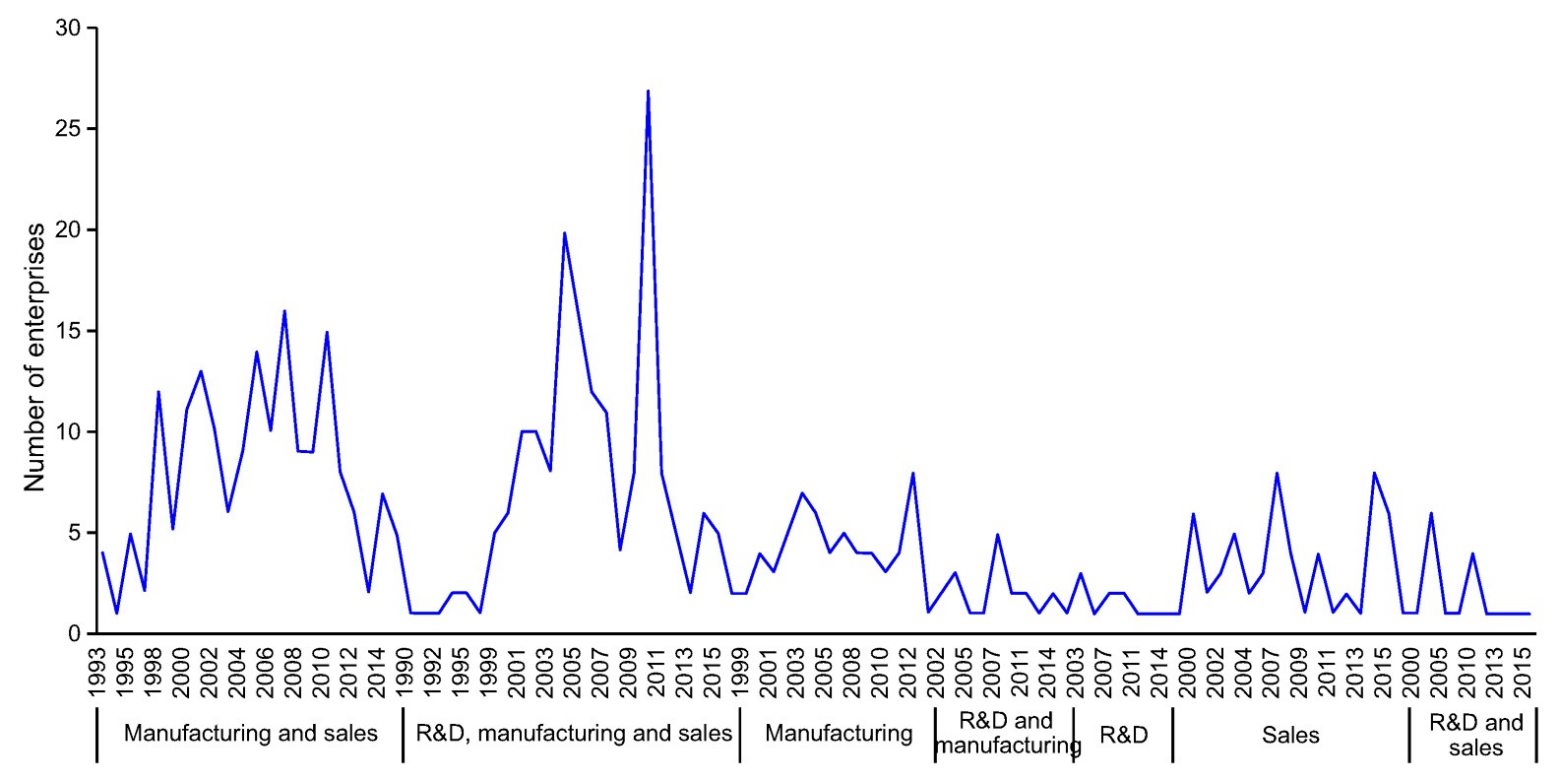
Temporal distribution of various value chain organization models of Taiwanese-listed OTC electronic information enterprises in Mainland China (annual increase in the number of enterprises).

Fig 5 outlines the temporal distribution of the organization models of Taiwanese OTC electronic information enterprises in Mainland China. The R&D–manufacturing–sales vertical integration model has been in place the longest (1990). The R&D, sales, and sales–R&D models (production sharing modularization) have the shortest history, only emerging after 1999. Enterprises focusing on R&D were founded later than those employing sales and R&D–sales models. For example, in 2003, Lite-On Technology Corporation and Pan-International Electronics founded subsidiaries (one in Beijing for the former, one each in Shenzhen and Zhengzhou for the latter) to design and develop computers, communications, digital home appliances, and network products, and provide related technical consultation.

In the modular network model of production expansion, enterprises engaging in manufacturing–sales were established between 1993 and 2015. For example, in 1993, Hon Hai Precision Industry Co. Ltd., Lite-On Technology Corporation, Delta Electronics Inc., and MiTAC Computing Technology Corp. founded subsidiaries in Shenzhen, Shanghai, Dongguan, and Foshan, respectively, to produce electronic components and related products and sell self-produced products and parts. Enterprises engaging in manufacturing and R&D–manufacturing were established in 1999 or later. For example, in 1999, Hon Hai Precision Industry Co. Ltd. established two subsidiaries in Shenzhen to produce electronic components, electronic devices, wire and cable assemblies, computer connectors, computer power supply molds and mold parts, and network system development technology information consultation. In 2002, Elitegroup Computer Systems and Chicony Electronics Co., Ltd. established subsidiaries in Shanghai and Shenzhen, respectively, for R&D and production of electronic products such as portable microcomputers, high-end servers, and their components.

The analysis shows that vertical integration has been the main organizational model of Taiwanese OTC electronic information enterprises in Mainland China, expanding from single production to the whole value chain in manufacturing, then sales, and finally R&D.

**Fig 6:**
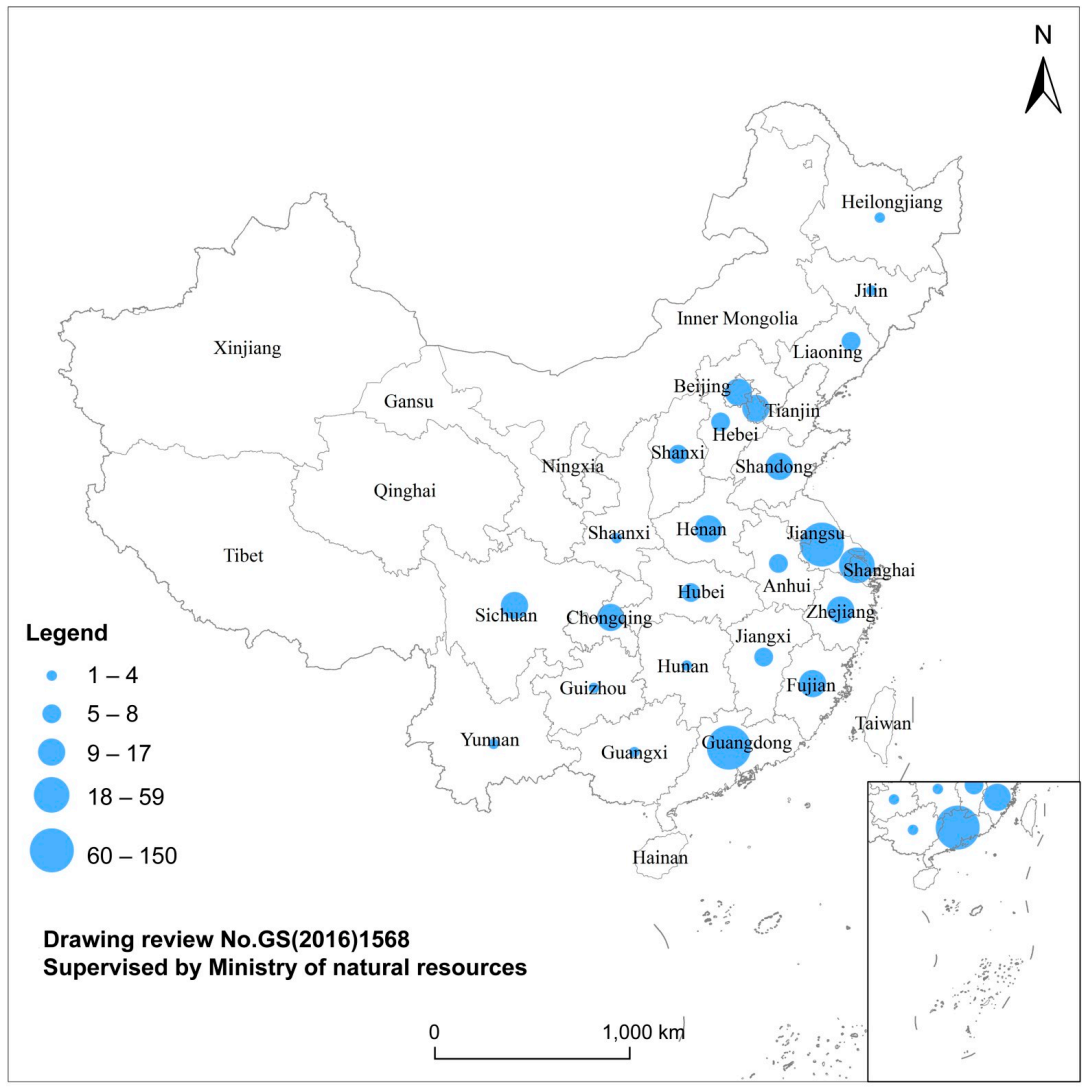
Spatial layout of Taiwanese-listed OTC electronic information enterprises in Mainland China.

There are five regional Taiwanese investment agglomerations in Mainland China (Fig 6), including two high agglomerations (the Yangtze River Delta represented by Jiangsu and the Pearl River Delta represented by Guangdong) and three medium agglomerations (the Bohai Economic Rim represented by Beijing, the Western Delta Economic Circle represented by Sichuan, and the Western Taiwan Straits Economic Zone represented by Fujian).

**Fig 7:**
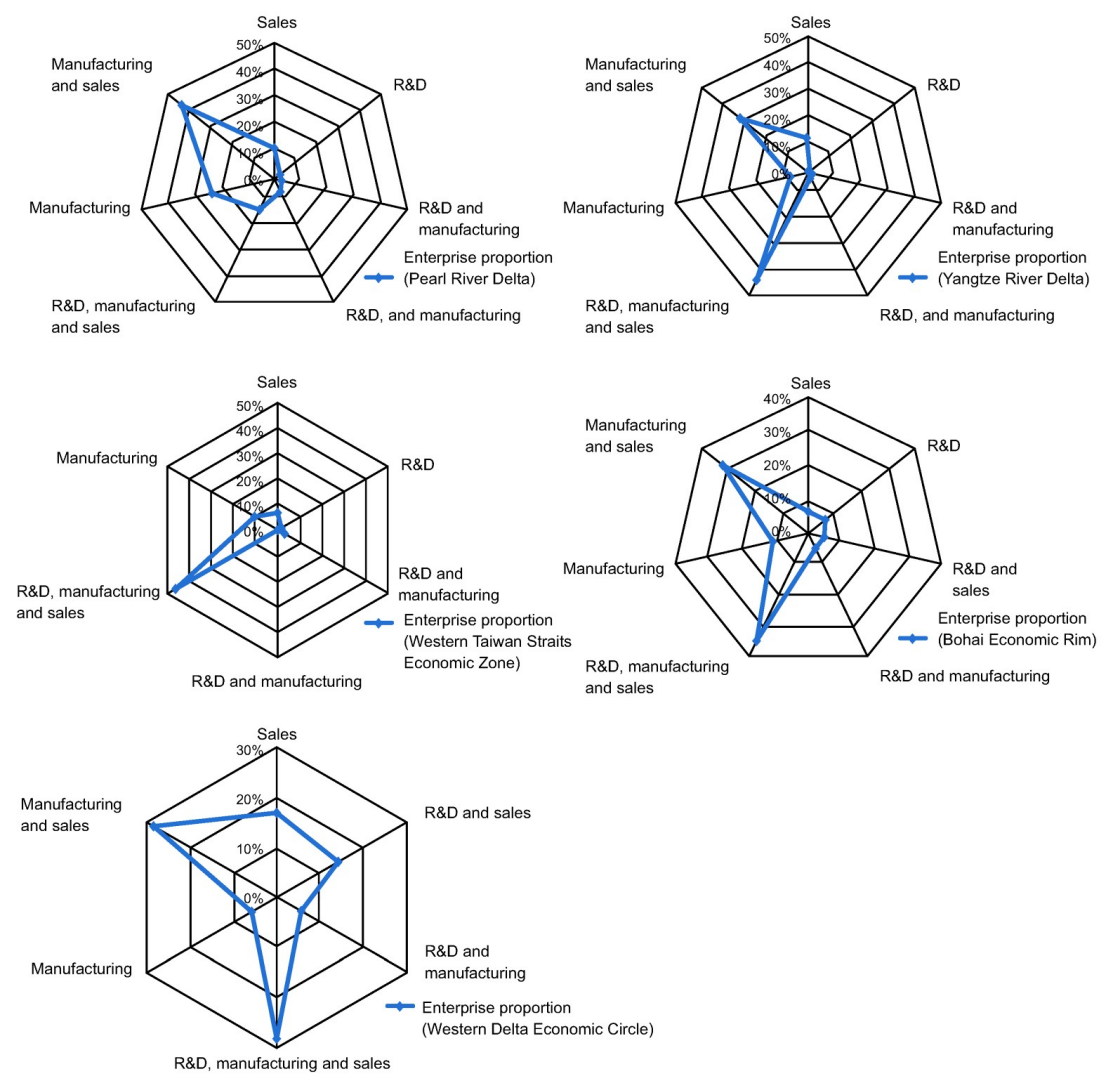
Layout characteristics of value chain models of enterprises in five Taiwanese investment agglomerations.

The value chain models in those regions are shown in Fig 7, which highlights the following:

There are seven value chain models in the Yangtze River Delta, Pearl River Delta, and Bohai Economic Rim, and six models in the Western Delta Economic Circle (no R&D model) and Western Taiwan Straits Economic Zone (no manufacturing–sales model).

Value chain models in the Yangtze River Delta, Bohai Economic Rim, and Western Delta Economic Circle showed a “double-winged” shape. The models accounting for a large proportion (denoted with double wings in Fig 7) were R&D–manufacturing–sales and manufacturing–sales. The value chain models in the Pearl River Delta and Western Taiwan Straits Economic Zone exhibited a “pendulum” shape, implying prominent development, and comprised manufacturing–sales and R&D–manufacturing–sales. Vertical integration remains the main value chain model in the Western Taiwan Straits Economic Zone, whereas the other four Taiwanese investment agglomerations have begun to expand to both ends of the production link (R&D and sales), but especially to the sales link.

**Fig 8:**
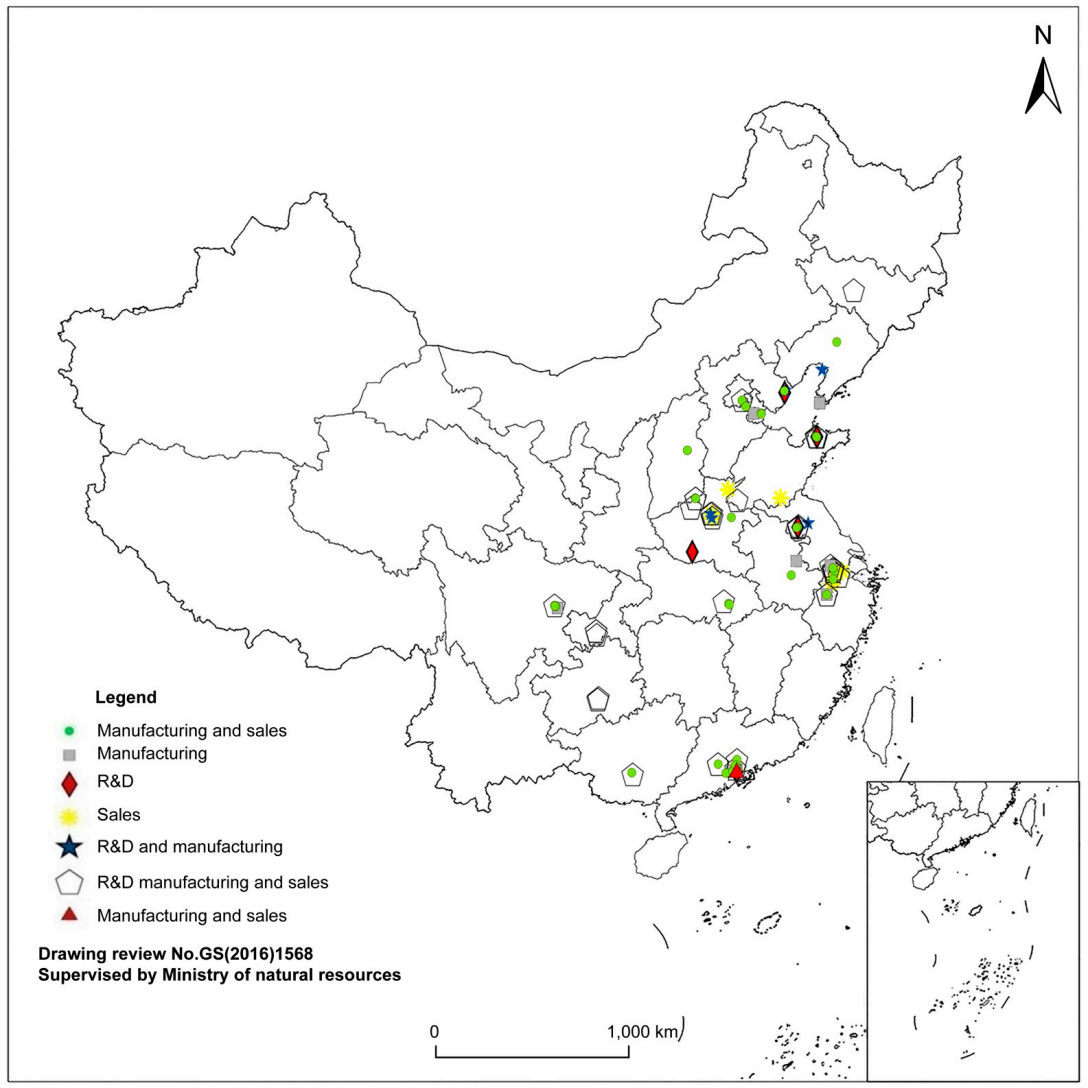
Spatial positioning map for the value chain models of enterprises (2016).

The spatial layout of enterprises employing different organization models varied at the city level, as shown in Fig 8, which highlights the following:

Most Taiwanese OTC enterprises engaging in the R&D–manufacturing–sales model were located in Shanghai and Suzhou, accounting for 24.3% and 12.7% of the total, respectively.

In the production sharing model, enterprises engaging in the sales model were concentrated in Shanghai, Shenzhen, and Suzhou, accounting for 25%, 21%, and 12% of the total, respectively. A few enterprises engaged in R&D and R&D–sales models; these tended to be located in inland areas. Enterprises engaged in the production sharing model were mainly located in Beijing, Shenzhen, Guangzhou, Zhengzhou, Shanghai, and so on, while enterprises engaging in R&D and R&D–sales models were mainly located in Beijing and Chongqing, followed by Shenzhen and Suzhou.

In the corporate network model of production expansion, enterprises engaging in the manufacturing–sales model had the widest spatial distribution, spreading to Guangdong, Jiangsu, Anhui, Sichuan, Shanxi, Hebei, Henan, Beijing, Tianjin, Hubei, and Hunan. The proportion of enterprises located in Suzhou, Shenzhen, Dongguan, and Shanghai, was more than 10% in each of those cities. The R&D–manufacturing model was adopted by five enterprises in Shenzhen, three in Shanghai, and two in Zhengzhou while the manufacturing model was adopted by 20 enterprises in Shenzhen and 11 in Suzhou.

In summary, high value-added enterprises adopting production sharing models (R&D, sales, and R&D–sales) were concentrated in Beijing, Shenzhen, and Suzhou, and tended to expand to inland cities such as Chongqing and Zhengzhou; enterprises adopting the manufacturing–sales product expansion model were the most widely distributed, covering the southeast coast and the central and western regions of Mainland China.

#### Micro level

Hon Hai Precision Industry Co. Ltd., Wistron Corp., and AsusTeK Computer Inc. strongly represented the three types of corporate organization models and therefore were selected from the List of Taiwanese Listed OTC Enterprises in Mainland China for microanalysis.

The representative enterprises for three organization models include: Hon Hai Precision Industry Co. Ltd. (founded in 1974), Wistron Corp. (founded in 1998), and AsusTeK Computer Inc. (founded in 1989). These enterprises are the largest and fastest growing international groups in the global computer, communication, and consumer electronics OEM field. As of 2016, they had established 129, 20, and 17 OTC electronic information manufacturing enterprises in Mainland China and mainly engaged in the organization models of production expansion, vertical integration, and production sharing, respectively (Table 1).

**Table 1:** Basic information of the representative enterprises.

Representative enterprise	Number of listed OTC electronic information manufacturing enterprises in Mainland China	Business scope	Corporate development strategy
Hon Hai Precision Industry Co., Ltd.	129	Design, production, and sales of key components and systems of high-tech products such as computers, network communications, and consumer electronics	Mold development + cost + supply chain
Wistron Corp.	20	R&D, production, and sales of notebook computers, desktop computers, servers, network home appliances, wired and wireless data communications, digital consumer electronics, and so on, and their components, spare parts, auxiliary parts, and so on.	Active diversification and integration of the upstream supply chain
AsusTeK Computer Inc.	17	Laptops, motherboards, graphics cards, servers, optical storage, wired/wireless network communication products, LCDs, personal digital assistants (PDAs), smart phones, and so on.	Challenging brand and OEM limits

A majority of Hon Hai Precision Industry’s OTC electronic information manufacturing enterprises in Mainland China adopted a model of production expansion, accounting for 54% of all its enterprises in Mainland China. This coincides with the group’s operating strategy of relying on its three major strengths of “mold development + cost + supply chain” to rapidly become a technology leader. Among the OTC manufacturers established by Wistron Corporation, those adopting the vertical integration model according to the group’s business philosophy of “creative diversification and upstream supply chain integration” accounted for 55% of the total. For AsusTeK Computer, 53% of its OTC enterprises formed a model of production sharing modularization. It is noteworthy that none of AsusTeK Computer’s enterprises adopted the manufacturing model, indicating that AsusTeK in Mainland China clearly favored high value-added production sharing. This is consistent with AsusTeK’s business strategy of “challenging brand and OEM limits” (i.e., AsusTeK launched its own brands, split its OEM business, and spun off Pegatron Corp).

**Fig 9:**
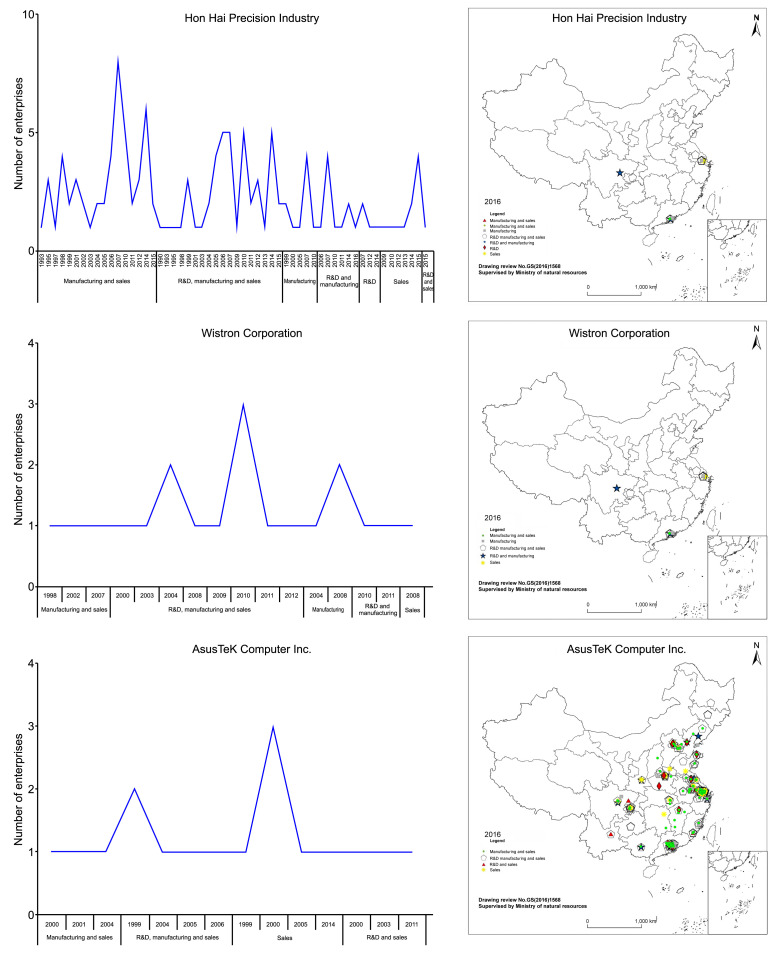
Spatiotemporal distribution of the value chain organization model of Hon Hai Precision Industry, Wistron Corporation, and AsusTeK.

Fig 9 shows the spatiotemporal evolution of the production organization models of the three representative enterprises’ OTC electronic information manufacturing subsidiaries in Mainland China.

From Fig 9, it is observed that the first enterprise is Hon Hai Precision Industry Co. Ltd. Since 1995, its subsidiaries in Mainland China have expanded production; beginning with the agglomerations in Guangdong and Jiangsu, the group began to spread to the inland areas of northern China such as Beijing, Tianjin, Shanxi, and Shandong. After 2007, high value-added subsidiaries adopting the R&D and sales production sharing models emerged and began to expand from Guangdong, Shandong, and Henan to coastal areas such as Shanghai and Zhejiang. However, newly established subsidiaries still mostly adopted production expansion models.

Wistron Corporation’s subsidiaries have emerged with a vertical integration of R&D, manufacturing, and sales since 2000. The number of new subsidiaries in a single year peaked at 3 in 2010 and has risen to a total of 20, which are mainly located in Suzhou and Zhongshan. In addition, since 2009 the group has begun to expand to Taizhou, Chongqing, and Qingdao.

AsusTeK Computer Inc. founded OTC subsidiaries in Mainland China after 1999. Beijing Asus Computer Co., Ltd. was AsusTeK’s earliest subsidiary in Mainland China and adopted a sales-oriented production-sharing model. From 2000 to 2006, subsidiaries adopting the vertical integration and production expansion models emerged and were concentrated in Suzhou and Shanghai. However, the subsidiaries established after 2010 have all adopted production-sharing models and have begun to spread to inland areas such as Chongqing. For example, Asus Computer (Chongqing) Co. Ltd. was established in 2011, with R&D–sales being the main organization model; Asus Digital International (Chongqing) Co. Ltd. was established in 2014, with sales being the main organization model.

The comparison of the three enterprises indicates that the development of enterprises using a production expansion model, represented by Hon Hai Precision, shows a state of continuous growth (Table 2). Even when such enterprises had subsidies engaging in production sharing models later, they still used production expansion as the development goal for strategic planning as the foundation for new subsidiaries. Enterprises using the product expansion model generally started earlier and witnessed the largest spread among the three organization models, that is, subsidiaries were established in the southeast coasts, central and western inland areas; however, the advantages of specialized division of labor are not apparent. Enterprises engaging in vertical integration, represented by Wistron, had high agglomerations and a relatively small spread. The enterprises were spread mainly in the Yangtze River Delta, Pearl River Delta, and Western Delta Economic Circle. Such enterprises were generally “big and complete, or small but complete,” with minimal economic efficiencies [63–65]. Enterprises engaging in a production-sharing model, represented by AsusTeK, were founded most recently and had high agglomerations. The enterprises were spread mainly in the Yangtze River Delta, Pearl River Delta, and Western Delta Economic Circle. In the early stage, most enterprises mainly focused on production expansion and vertical integration. Later, however, production sharing developed rapidly and was characterized by a clear advantage of specialized division of labor and economic efficiency.

**Table 2:** Comparison of the three organization models.

	Production expansion	Vertical integration	Production sharing modularization
Development time	Earlier	Earliest	Late
Characteristics	Such enterprises developed stably and began to gradually turn to the production sharing models. Representative enterprises had the largest spread, both along the southeast coasts and in central and western inland areas.	Such enterprises were big and complete, or small but complete and had high agglomerations. Representative enterprises were mainly spread throughout the Yangtze River Delta, Pearl River Delta, and Western Delta Economic Circle.	Such enterprises had high agglomerations. Representative enterprises were mainly spread throughout the Yangtze River Delta, Pearl River Delta, and Western Delta Economic Circle. In the early stage, they usually adopted production expansion and vertical integration models.
Advantages	Stable development	Comprehensive development	Clear advantage in specialized division of labor, high income, and rapid development
Disadvantages	The advantage of specialized division of labor was subtle.	The advantage of specialized division of labor was hardly visible.	N/A
Representative enterprises	Hon Hai Precision Industry, Delta, Quanta, Foxlink Group, etc.	Wistron, Clevo, Coretronic, and Catcher	AsusTeK, Inventec Appliances Corp., and Pan-International Electronics

## Discussion

Based on the core idea of value chain theory from the perspective of enterprise spatial organization, we combined ArcGIS spatial visualization and case analyses to investigate the characteristics and evolution of the spatial organization of the Taiwanese-listed OTC electronic information enterprises in Mainland China, focusing on typical Taiwanese enterprises in Taiwanese investment agglomerations. Results show that vertical integration is the main production organization model of Taiwanese-listed OTC electronic information enterprises in Mainland China. This model still prevails in the Western Taiwan Straits Economic Zone, whereas the other four Taiwanese agglomerations in the Bohai Economic Rim, Yangtze River Delta, Pearl River Delta, and Western Delta Economic Circle have begun to expand to both ends of the production link, especially to the sales link. High value-added enterprises adopting production sharing models (R&D, sales, and R&D–sales models) have begun to shift to inland cities in central and western China such as Chongqing and Zhengzhou. Enterprises adopting the manufacturing–sales model (a production expansion model) were the most widely distributed.

Compared with the organizational characteristics of all foreign multinational corporations in China [66–68], the results showed that Taiwanese enterprises have expanded in Mainland China from the single production function to sales, R&D, and other links. The value chain fragmentation layouts of Taiwanese electronic information enterprises matched the Chinese city hierarchy. For example, high value-added links were concentrated not only in first-tier cities such as Beijing, Shanghai, Shenzhen, and Guangzhou, but have begun to spread to regional central and provincial capital cities such as Chongqing and Zhengzhou. The Yangtze River Delta and Bohai Economic Rim were common core areas for multinational enterprises from developed countries and Taiwanese enterprises looking to expand into both ends of the production link. However, the Western Delta Economic Circle was a pioneer in attracting Taiwanese enterprises and has become an important destination for Taiwanese electronic information enterprises to set up R&D and sales links.

The main reason for the Western Delta Economic Circle, especially Chongqing, being so successful is due to its a favorable policy toward Taiwan, and since the average wages in the manufacturing sector are close to the national average. Zhengzhou, located in the midlands, has a similar labor cost advantage and scientific research potential, in addition to the geographical advantage of being connected to both the east and west of the country. In such a circumstance, a policy pertaining to targeted industrial cooperation can be formulated to improve the ITE industry’s sustainability for both Mainland China and Taiwan by integrating the comparative advantages of other economic zones.

1. The Bohai Rim region: There is active innovation, frequent technology exchanges, it leads in terms of technology market turnover and innovation factors, and the average wages of the manufacturing and software industries, as well as industrial land prices, are higher than in the Yangtze River Delta. However, it has not become a hub for Taiwanese investment. This area could strengthen cooperation in high-value fields, such as the software design industry, the operation centers of finished machine products, and R&D centers, by attracting Taiwanese investment.
2. The Yangtze River Delta area: This area is defined by an active capital market, an atmosphere conducive for innovation, a broad consumer market, low taxes, robust industrial support, research and development, manufacturing, sales, and development of ecological settlements. However, with the increase in labor wages and land prices, it is necessary to regularly strengthen the localization of Taiwanese enterprises by seeking to build technical standards in collaboration with local governments and enterprises, jointly develop and design products in line with the mainland market, and establish special distribution channels and marketing outlets.
3. The Pearl River Delta area: This area has unique geographical advantages, as it is adjacent to Hong Kong and Macao. It has the lowest level of “foreign-funded” tax burdens and a variety of preferential policies for Taiwanese business ventures. However, it lags behind the Bohai Rim and Yangtze River Delta with respect to scientific research institutions and higher education. Therefore, it is necessary to improve the regional innovation environment and the technological research and development ability of Taiwanese enterprises and expand the domestic market. Under appropriate policy guidance, Taiwanese enterprises in Shenzhen, Guangzhou, and other central cities should strengthen localization in the Pearl River Delta and develop high value-added relationships (e.g., software design and marketing). Labor-intensive Taiwan-funded industries should be transferred to the lower-cost northwest Guangdong Province regions, such as Heyuan, Zhanjiang, Qingyuan, and other cities.
4. The Western Taiwan Straits Economic Zone: With the advantages of lower labor costs, scientific research, and a high talent pool, as well as the preferential local government policies aimed at promoting transportation construction and subsidizing of manufacturers’ operating costs, this area is expected to undertake the industrial transformation for the east coast and become a new Taiwanese-funded computer production hub in the future. In the interim, the area should attract Taiwanese investment from upstream and downstream enterprises to continuously improve the industrial VC and expand the market potential in the central and western regions of Mainland China.

This study has several limitations. First, due to data availability, the sample only included the data of Taiwanese OTC electronic information enterprises from 1990 to 2016. If the data of unlisted Taiwanese electronic information enterprises in Mainland China is included, the sample will increase greatly. Nonetheless, the study may remain limited by the small number of subsidiaries. Second, the organizational models of Taiwanese enterprises in Mainland China and their spatiotemporal evolution characteristics were analyzed from the value chain perspective. Subsequent research could integrate the VCs in the manufacturing industry and productive services industry, as well as the relationships between enterprises and non-enterprise actors. Geographic information system mapping could be used together with social network analysis to further reveal the organizational mechanism and embedding path of Taiwanese enterprises in Mainland China.
